# Theoretical and analyzed data related to thermal degradation kinetics of poly (L-lactic acid)/chitosan-*grafted*-oligo L-lactic acid (PLA/CH-*g*-OLLA) bionanocomposite films

**DOI:** 10.1016/j.dib.2016.11.100

**Published:** 2016-12-07

**Authors:** Akhilesh Kumar Pal, Vimal Katiyar

**Affiliations:** Department of Chemical Engineering, Indian Institute of Technology Guwahati, Guwahati, Assam 781039, India

**Keywords:** Poly (lactic acid), Chitosan, Isoconversional method, Thermal degradation kinetics, Activation energy

## Abstract

The theoretical and analyzed data incorporated in this article are related to the recently published research article entitled “Thermal degradation behaviour of nanoamphiphilic chitosan dispersed poly (lactic acid) bionanocomposite films” (http://dx.doi.org/10.1016/j.ijbiomac.2016.11.024) (A.K. Pal, V. Katiyar, 2016) [1]. Supplementary information and data (both raw and analyzed) are related to thermal degradation kinetics and explains various model fitting and is conversional methods, which are used in this research work to enhance the knowledge about degradation behaviour of PLA/CH-*g*-OLLA bionanocomposite system. Non-isothermal degradation kinetics of such polymeric system was proposed using Kissinger, Kissinger–Akahira–Sunose, Flynn–Wall–Ozawa and Augis and Bennett models to estimate the activation energies (*E_a_*) and *R*^2^ values.

**Specifications Table**

**Table:** 

Subject area	Material Sciences
Morespecificsubjectarea	Polymer degradation
Type of data	Text file, equations and tables
How data was acquired	Data is analyzed using Thermogravimetric analyzer (Libra TG 209, Netzsch, Germany) under the following condition: Sample weight = ~5–8 mg, Temperature range = 35–650 °C, Heating rates = 2, 5 and 10 °C min^−1^ and Atmosphere = inert atmosphere (flow rate ~50 mL min^−1^)
Data format	Text and analyzed data
Experimental factors	PLA and PLA/CH-*g*-OLLA bionanocomposite films were fabricated by varying CH-*g*-OLLA loading from 1–5% (wt/wt).
Experimental features	Various combinations of PLA/CH-*g*-OLLA bionanocomposite films were prepared by solution casting method. The activation energies (*E_a_*) and *R*^2^ values for all the combinations were analyzed by the data obtained from thermogravimetric analysis for evaluating the degradation behaviour at each conversion.
Data source location	Department of Chemical Engineering, Indian Institute of Technology Guwahati, Guwahati, Assam-781039 (India)
Data accessibility	Data are presented in this article only

**Value of the data**

• The basic information about thermal degradation kinetics is required to understand the degradation behaviour of any polymeric system.
• Various model fitting and isoconversional methods give information about the degradation pathway and related analyses confirm the degraded products. Both parameters are useful to check the industrial viability and recyclability of PLA/CH-*g*-OLLA bionanocomposites.
• The presented basic information can be useful for other researchers to choose suitable model on the basis of their assumptions and limitations.
• The activation energies with *R*^2^ values are calculated using Flynn–Wall–Ozawa and Kissinger–Akahira–Sunose model.

## 1 Basic theoretical data

### 1.1 Theoretical consideration of thermal degradation kinetics

A general reaction is applied to describe the thermal decomposition of polymeric materials as mentioned in Eq. (S1).

(S1) $$P\left(\frac{\mathrm{solid}}{\mathrm{liquid}}\right)\to Q\left(\frac{\mathrm{solid}}{\mathrm{liquid}}\right)+R\left(\mathrm{volatile or gases}\right)$$

where, *P* and *Q* are the reactants and *R* is the reaction product generated during the consumption of *P* and *Q*.

The decomposition rate ($\frac{d\alpha }{\mathrm{dt}}$), for isothermal reactions may be described as shown in Eq. (S2).

(S2) $$\frac{d\alpha }{\mathrm{dt}}=k\left(T\right)f\left(\alpha \right)$$

where, $\alpha =\mathrm{Extent of conversion}=\frac{W_{0}-W_{t}}{W_{0}-W_{f}}$, *W*_0_, *W_t_* and *W_f_* are initial weight, weight at time *t* and final weight of the sample respectively. *k*(*T*)is the rate constant at temperature *T*. f(α) is a function of reaction model, which depends on degradation mechanism. The temperature dependant rate constant may be explained with the help of Arrhenius equation as expressed in Eq. (S3).

(S3) $$k\left(T\right)=A\exp\left(-\frac{E_{a}}{\mathrm{RT}}\right)$$

where, *E_a_*, *A*, *T* and *R* are the activation energy (kJ mol^−1^), pre-exponential factor (s^−1^), absolute temperature (K) and universal gas constant (8.314 J mol^−1^ K^−1^) respectively. The above Eqs. (S2), (S3) can be combined together and formed Eq. (S4).

(S4) $$\frac{d\alpha }{\mathrm{dt}}=A\exp\left(-\frac{E_{a}}{\mathrm{RT}}\right)f\left(\alpha \right)$$

The decomposition rate ($\frac{d\alpha }{\mathrm{dt}}$) is described for non-isothermal reactions with a heating rate ($\;\beta =\frac{\mathrm{dT}}{\mathrm{dt}}$) and as expressed in Eq. (S5).

(S5) $$\frac{d\alpha }{\mathrm{dT}}=A\frac{1}{\beta }\exp\left(-\frac{E_{a}}{\mathrm{RT}}\right)f\left(\alpha \right)$$

where, $\frac{d\alpha }{\mathrm{dT}}$ is the rate of non-isothermal reactions at β heating rate. The pre-exponential factor and activation energy have been calculated by the rate equations only if f(α) remains unchanged till the completion of the reaction, which is barely feasible in solid state reactions [2]. The thermal degradation of polymeric samples is highly complicated so it is considered that the decomposition reaction follows a simple reaction of nth order. Hence, f(α) is expressed in the form of ${\left(1-\alpha \right)}^{n}$, where, *n* denotes the reaction order. In the case of gas and liquid kinetics, the collision and energy barrier concepts are generally correlated with Arrhenius constants, which can be directly represented by the kinetic parameters calculated from TGA data. However, solid state kinetics do not based on the same consideration. Hence, in this case, the activation energy is illustrated as the average excess energy recovered from the vibration of atoms or molecules at a prescribed temperature. This activation energy is also termed as apparent activation energy, which is also associated to the cleavage of chemical bonds. Different approaches are suggested to calculate the kinetic constants by using the TGA data, which are obtained from isothermal and non-isothermal conditions. Such approaches or methods are proposed to resolve the above equations by differentiation, integration and approximation. On that basis, models are majorly divided into two types i.e. Isoconversional methods and model fitting methods [3].

## 2 Isoconversional methods

Isoconversional methods are recognized as the most appropriate methods to calculate the activation energy of the reactions, which are thermally active. Such methods are used to determine the *E_a_* value at different conversions (*α*) without any modelistic assumptions [4]. The previous knowledge of thermal decomposition mechanism is not required in isoconversional methods. According to the isoconversional principle, the rate of reaction is a function of temperature at a constant conversion value. Such models are best applicable for multiple heating rate values [5].

### 2.1 Kissinger–Akahira–Sunose Model

It is an integral isoconversional method, which is based on the Murray and White approximation for temperature integral and is mentioned in Eq. (S6).

(S6) $$\frac{d\alpha }{f(\alpha )}=\frac{A}{\beta }\exp\left(-\frac{E_{a}}{\mathrm{RT}}\right)\mathrm{dT}$$

Integrate Eq. (S6) with the initial boundary condition of α=0 at $T=T_{0}$ and the obtained expression is mentioned in Eq. (S7).

(S7) $$g\left(\alpha \right)=\int_{0}^{\alpha }\frac{d\alpha }{f(\alpha )}=\frac{A}{\beta }\int_{T_{0}}^{T}\exp\;(-\frac{E_{a}}{\mathrm{RT}})\mathrm{dT}\equiv \frac{AE_{a}}{\beta R}p\left(\frac{E_{a}}{\mathrm{RT}}\right)$$

(S8) $$p\left(\frac{E_{a}}{\mathrm{RT}}\right)≅\frac{\exp\left(-\frac{E_{a}}{\mathrm{RT}}\right)}{{\left(\frac{E_{a}}{\mathrm{RT}}\right)}^{2}}$$

The essential assumption of this technique is that the $A,\;E_{a}$ and f(α) are temperature independent terms. However, *A* and *E_a_* are also independent of *α*. The term g(α) represents a kinetic model in integral form. Eq. (S7) is integrated followed by logarithms on both sides and final expression is obtained as in Eq. (S9).

(S9) $$\ln g\left(\alpha \right)=\ln\left(\frac{AE_{a}}{R}\right)-\ln\beta +\ln p(\frac{E_{a}}{\mathrm{RT}})$$

Eq. (S9) is further solved and the final expression is shown in Eq. (S10).

(S10) $$\ln\frac{\beta }{T^{2}}=\ln\left(\frac{\mathrm{AR}}{E_{a}g\left(\alpha \right)}\right)-\frac{E_{a}}{\mathrm{RT}}$$

The activation energy for each degree of conversion (*α*) is calculated by the slope of linear curve of $\ln\frac{\beta }{T^{2}}$ versus $-\frac{1}{T}$. The value of pre-exponential factor (*A*) is obtained by the intercept of the same linear curve [6].

### 2.2 Flynn–Wall–Ozawa Model

Flynn–Wall–Ozawa model is also a well-established integral isoconversional model [3]. The important assumption in this model is that the conversion function f(α) is independent with the alteration in heating rate for all values of the degree of conversion *α*. It follows Arrhenius type temperature dependence without any assumptions related to the form of the kinetic equation. In this model, the measurement of temperature with respect to fixed values of *α* is observed at different heating rates β from experiments [7]. Flynn–Wall–Ozawa method is relatively a simple method to calculate the value of activation energy from curve of weight loss versus temperature at different heating rates.

Integrate Eq. (S6) with the condition of $\alpha =\alpha_{0}$ to $\alpha =\alpha_{p}$ on both sides and the obtained expression is shown in Eq. (S11).

(S11) $$g\left(\alpha \right)=\int_{\alpha_{0}}^{\alpha_{p}}\frac{d\alpha }{f(\alpha )}=\frac{A}{\beta }\int_{\alpha_{0}}^{\alpha_{p}}\exp(-\frac{E_{a}}{\mathrm{RT}})\mathrm{dT}$$

It is assumed that $x=\frac{E_{a}}{\mathrm{RT}}$ and now integrate the right hand side of Eq. (S11), which converts in Eq. (S12).

(S12) $$g\left(\alpha \right)=\int_{\alpha_{0}}^{\alpha_{p}}\frac{d\alpha }{f(\alpha )}=\frac{AE_{a}}{\beta R}p\left(x\right)$$

Taking logarithms on both sides and after re-arranging, the obtained expression is mentioned in Eq. (S13).

(S13) $$\log\;\beta =\log\frac{AE_{a}}{g\left(\alpha \right)R}+\log p\left(x\right)$$

The doyle׳s approximation is used for simplification of Eq. (S13) and the obtained Eq. is shown in Eq. (S14).

(S14) logp(x)=−2.315−0.4567x

Substituting Eq. (S14) into Eq. (S13) and final form of Flynn–Wall–Ozawa model equation is obtained as in Eq. (S15).

(S15) $$\log\beta =\log\frac{AE_{a}}{g\left(\alpha \right)R}-2.315-\frac{0.4567E_{a}}{\mathrm{RT}}$$

The values of activation energy and pre-exponential factor are determined from the slope $\left(\frac{0.4567E_{a}}{R}\right)$ and intercept $\left(\log\frac{AE_{a}}{g\left(\alpha \right)R}-2.315\right)$ respectively, by plotting a linear curve between logβ and $\left(-\frac{1}{T}\right)$ at constant value of fractional conversion [8]. Basically, Flynn–Wall–Ozawa model is used only for those polymeric systems in which more than one reactions are taking place simultaneously in such a way that the activation energy changes with respect to time. On the other hand, this model becomes fail, when extensively different type of reactions are occurring simultaneously [9].

### 2.3 Augis and Bennett model

Augis and Bennett is proposed to be an isoconversional model, which shows the dependence of temperature on heating rate and is also used to calculate the kinetics parameters by using Eq. (S16).

(S16) $$\ln\left(\frac{\beta }{T_{m}-T_{0}}\right)=\ln A-\frac{E_{a}}{{\mathrm{RT}}_{m}}$$

where, $T_{m}$ and *T*_0_are the temperature at maximum degradation and the onset temperature of the DTG curve respectively. *E_a_* and *R* are calculated from the slope $\left(\frac{E_{a}}{R}\right)$ and intercept (lnA) respectively, by plotting a linear curve between $\ln\left(\frac{\beta }{T_{m}-T_{0}}\right)$ and $\left(-\frac{1}{T_{m}}\right)$ [10].

## 3 Model fitting methods

Model fitting methods are based on the different models fittings at a particular temperature obtained from DTG curve and also determine the values of activation energy and pre-exponential factor. Many non-isothermal model fitting methods are available but Kissinger model is the most popular among all [11].

### 3.1 Kissinger model

This model fitting method is widely used for calculating kinetic triplets (*E_a_*, *A* and *R*) and is based on the peak degradation temperature, obtained from the DTG thermogram. The activation energy is calculated at maximum temperature from DTG curve. It should be very well understood that Kissinger does not belong to isoconversional method because the fractional conversion at degradation peak and peak temperature should change with change in heating rates [12]. This method may also be applied to calculate activation energy without prior knowledge of reaction mechanism [7], [13]. According to the principle of maxima and minima, the conversion rate derivative should be equal to zero at peak temperature. In this way, Eq. (S6) can be re-written in the form of Eq. (S17).

(S17) $$\frac{d\alpha }{\mathrm{dT}}=A\frac{1}{\beta }\exp\left(-\frac{E_{a}}{\mathrm{RT}}\right){\left(1-\alpha \right)}^{n}$$

One assumption is made that the rate of reaction is maximum at the peak temperatures (*T*_max_), so the conversion rate derivative at *T*_max_ becomes zero and the obtained expression is mentioned in Eq. (S18).

(S18) $$\frac{d}{\mathrm{dT}}\left(\frac{d\alpha }{\mathrm{dT}}\right)=\frac{d}{\mathrm{dT}}\left[\frac{A}{\beta }\exp\left(-\frac{E_{a}}{\mathrm{RT}}\right){\left(1-\alpha \right)}^{n}\right]$$

By putting $\frac{d^{2}\alpha }{dT^{2}}=0$ at *T*=*T_m_* and re-arranging then the Eq. (S18) can be expressed in the form of Eq. (S19).

(S19) $$\frac{E_{a}\beta }{R{T_{m}}^{2}n{\left(1-\alpha \right)}^{n-1}}=A\exp\left(-\frac{E_{a}}{RT_{m}}\right)$$

where, *T_m_* is the temperature at which rate of decomposition is maximum. Assume, the order of degradation is one i.e. *n*=1 then Eq. (S19) converts into Eq. (S20).

(S20) $$\frac{\beta }{{T_{m}}^{2}}=\frac{\mathrm{AR}}{E}\exp\left(-\frac{E_{a}}{RT_{m}}\right)$$

Apply logarithms on both sides and obtained final expression is shown in Eq. (S21).

(S21) $$\ln\frac{\beta }{{T_{m}}^{2}}=\ln\frac{\mathrm{AR}}{E}-\frac{E_{a}}{RT_{m}}$$

This Eq. (S21) is known as famous “Kissinger” equation. *E_a_* and *R* are determined by the slope (*R*) and intercept $\left(\ln\frac{\mathrm{AR}}{E}\right)$ respectively, by plotting a linear curve between $\left(\ln\frac{\beta }{{T_{m}}^{2}}\right)$ and $\left(-\frac{1}{T_{m}}\right)$. If the curve of rate of reaction is free from shoulders and activation energy is almost consistent over the full range of conversion, it means that the thermal decomposition process is controlled by a single step model. But the variation in activation energy with conversion is much obvious and is controlled with many possible processes involved during thermal degradation. Normally, the activation energy increases with increase in conversion [14]. Some limitations are also associated with this method. The first limitation is based on the fact that the reaction model function (f(α)) should not depend on the heating rate to calculate the activation energy values. The second limitation is that Kissinger method is able to produce only one activation energy value for any process without the knowledge of its actual kinetic complexity. So, it always denotes only a single step kinetics. However, more than one value of activation energy is required for multi-step kinetics. Hence, the support of isoconversional methods is required to verify kinetic parameter values calculated by Kissinger model [15].

### 3.2 Analyzed data

The reported data incorporates the essential information about the degradation pathway of PLA and PLA/CH-*g*-OLLA bionanocomposite films in relation with activation energies at various conversion, which are analyzed with the help of isoconversional models such as Flynn–Wall–Ozawa and Kissinger–Akahira–Sunose by using Eqs. (S15) and (S10) respectively as shown in Table 1. Simultaneously, *R*^2^ values at each conversion for various heating rates are also calculated to check the applicability of isoconversional models as shown in Table 2.

## 4 Experimental design, materials and methods

PLA and PLA/CH-*g*-OLLA bionanocomposite films were prepared by well-known solution casting method with the help of various combinations of PLA and CH-*g*-OLLA bionanocomposite [1].

### 4.1 Thermogravimetric analysis

Thermogravimetric analyzer (Libra TG 209, NETZSCH, Germany) was used to observed the degradation behaviour of PLA/CH-*g*-OLLA bionanocomposite films (~5–8 mg) at temperature range from 35–650 °C at various heating rates such as 2, 5 and 10 °C min^−1^ under inert atmosphere (flow rate ~50 mL min^−1^). The collected raw data were used to measure activation energies (*E_a_*) and *R*^2^ values at each conversion using Flynn–Wall–Ozawa and Kissinger–Akahira–Sunose models.

## Figures and Tables

**Table 1:** Calculated values of activation energies (*E_a_*) at various conversions using Flynn–Wall–Ozawa and Kissinger–Akahira–Sunose models.

**Conversion (α)**	**Flynn–Wall–Ozawa**				**Kissinger–Akahira–Sunose**			
	**NPLA**	**PLA/CH-*****g*****-OLLA (1%)**	**PLA/CH-*****g*****-OLLA (3%)**	**PLA/CH-*****g*****-OLLA (5%)**	**NPLA**	**PLA/CH-*****g*****-OLLA (1%)**	**PLA/CH-*****g*****-OLLA (3%)**	**PLA/CH-*****g*****-OLLA (5%)**
0.1	258.14	265.42	170.07	102.55	261.47	269.35	169.42	98.73
0.2	258.99	270.97	202.5	106.76	262.17	275.04	203.19	102.74
0.3	259.5	273.05	216.83	107.89	262.61	277.08	218.11	103.76
0.4	260.94	248.47	212.61	107.39	264.04	251.13	213.55	103.1
0.5	259.63	248.32	236.29	107.76	262.6	250.89	238.35	103.4
0.6	258.56	250.04	217.76	107.76	261.41	252.66	218.79	103.32
0.7	253.7	228.3	198.31	103.09	256.25	229.74	198.29	98.39
0.8	258.52	232.21	203.68	97.35	261.24	233.76	203.84	92.27
0.9	245.15	233.83	223.58	107.99	249.75	235.4	224.67	103.34

**Table 2:** Calculated values of *R*^2^ at various conversions using Flynn–Wall–Ozawa and Kissinger–Akahira–Sunose models.

**Conversion (α)**	**Flynn–Wall–Ozawa**				**Kissinger–Akahira–Sunose**			
	**NPLA**	**PLA/CH-*****g*****-OLLA (1%)**	**PLA/CH-*****g*****-OLLA (3%)**	**PLA/CH-*****g*****-OLLA (5%)**	**NPLA**	**PLA/CH-*****g*****-OLLA (1%)**	**PLA/CH-*****g*****-OLLA (3%)**	**PLA/CH-*****g*****-OLLA (5%)**
0.1	0.994	0.999	0.998	0.998	0.994	0.999	0.998	0.998
0.2	0.996	0.998	0.998	0.998	0.996	0.998	0.998	0.998
0.3	1	0.999	0.998	0.999	1	0.999	0.997	0.999
0.4	0.998	0.999	0.997	0.998	0.998	0.999	0.997	0.997
0.5	0.999	1	0.998	0.998	0.998	1	0.998	0.997
0.6	0.998	0.999	0.997	0.997	0.999	0.999	0.997	0.996
0.7	0.999	0.999	0.999	0.998	0.998	0.999	0.999	0.997
0.8	0.997	0.999	0.998	0.998	0.998	0.999	0.997	0.998
0.9	0.998	0.999	0.999	0.998	0.997	0.999	0.999	0.998
