# Supplementary material for: Theoretical and analyzed data related to thermal degradation kinetics of poly (L-lactic acid)/chitosan-grafted-oligo L-lactic acid (PLA/CH-g-OLLA) bionanocomposite films
Source: Data Brief. 2016 Dec 7;10:304–11. doi: 10.1016/j.dib.2016.11.100 (PMC5156601; doi:10.1016/j.dib.2016.11.100)
Supplement: Supplementary file 1 — Supplementary material [file mmc1.docx]

November 19, 2016

To,

**Professor Hao-Ran Wang,**

**Editor-in-Chief,**

**Data in Brief, Elsevier**

Subject: Revised submission of a manuscript for possible publication in *Data in Brief, Elsevier.*

Dear Prof. Hao-Ran Wang,

Please find the attached revised manuscript titled, *“****Theoretical Data Analysis for Thermal Degradation Kinetics of Poly (L-lactic acid)/Chitosan-grafted-oligo L-lactic acid (PLA/CH-g-OLLA) Bionanocomposite Films****”* (Manuscript Id: DIB-D-16-00857) for consideration as an article in *Data in Brief, Elsevier*. This manuscript reports original work by Akhilesh Kumar Pal and Vimal Katiyar from Indian Institute of Technology Guwahati, India. The corresponding author for this publication is Dr. Vimal Katiyar ([vkatiyar@iitg.ernet.in](mailto:vkatiyar@iitg.ernet.in)).

The theoretical and analyzed data incorporated in this article are related to the recently published research article entitled “Thermal degradation behaviour of nanoamphiphilic chitosan dispersed poly (lactic acid) bionanocomposite films” (http://dx.doi.org/10.1016/j.ijbiomac.2016.11.024) [1]. The supplementary information and data (both raw and analyzed) are related to thermal degradation kinetics and explains various model fitting and is conversional methods, which are used in this research work to enhance the knowledge about degradation behaviour of PLA/CH-*g*-OLLA bionanocomposite system. Non-isothermal degradation kinetics of such polymeric system was proposed using Kissinger, Kissinger-Akahira-Sunose, Flynn-Wall-Ozawa and Augis & Bennett models to estimate the activation energies (E_a_) and R^2^ values.

This manuscript, or its contents in any other form, has not been published previously by any of the authors and/or is not under consideration for publication in another journal at the time of submission. All the authors have seen and approved the submission of the manuscript in this journal.

Thank you for considering our manuscript.

Sincerely,

Dr. Vimal Katiyar
